# Direct Visualization of Mucus Production by the Cold-Water Coral *Lophelia pertusa* with Digital Holographic Microscopy

**DOI:** 10.1371/journal.pone.0146766

**Published:** 2016-02-03

**Authors:** Eva-Maria Zetsche, Thierry Baussant, Filip J. R. Meysman, Dick van Oevelen

**Affiliations:** 1 Analytical, Environmental & Geo-Chemistry, Vrije Universiteit Brussel, Brussels, Belgium; 2 Department of Ecosystem Studies, Royal Netherlands Institute for Sea Research (NIOZ-Yerseke), Yerseke, The Netherlands; 3 International Research Institute of Stavanger (IRIS), Randaberg, Norway; King Abdullah University of Science and Technology, SAUDI ARABIA

## Abstract

*Lophelia pertusa* is the dominant reef-building organism of cold-water coral reefs, and is known to produce significant amounts of mucus, which could involve an important metabolic cost. Mucus is involved in particle removal and feeding processes, yet the triggers and dynamics of mucus production are currently still poorly described because the existing tools to study these processes are not appropriate. Using a novel microscopic technique—digital holographic microscopy (DHM)–we studied the mucus release of *L*. *pertusa* under various experimental conditions. DHM technology permits μm-scale observations and allows the visualization of transparent mucoid substances in real time without staining. Fragments of *L*. *pertusa* were first maintained in flow-through chambers without stressors and imaged with DHM, then exposed to various stressors (suspended particles, particulate food and air exposure) and re-imaged. Under non-stressed conditions no release of mucus was observed, whilst mucus strings and sheaths were produced in response to suspended particles (activated charcoal and drill cuttings sediment) i.e. in a stressed condition. Mucus strings and so-called ‘string balls’ were also observed in response to exposure to particulate food (brine shrimp *Artemia salina*). Upon air-exposure, mucus production was clearly visible once the fragments were returned to the flow chamber. Distinct optical properties such as optical path length difference (OPD) were measured with DHM in response to the various stimuli suggesting that different mucus types are produced by *L*. *pertusa*. Mucus produced to reject particles is similar in refractive index to the surrounding seawater, suggesting that the energy content of this mucus is low. In contrast, mucus produced in response to either food particle addition or air exposure had a higher refractive index, suggesting a higher metabolic investment in the production of these mucoid substances. This paper shows for the first time the potential of DHM technology for the detection, characterization and quantification of mucus production through OPD measurements in *L*. *pertusa*.

## Introduction

*Lophelia pertusa* is the most common scleractinian cold-water coral (CWC) and these sessile filter-feeders form extensive reef frameworks on the seafloor, mostly at a depth range of 300 to 800 m [1,2]. These CWC reefs are hotspots of biodiversity [2] and organic matter processing [3,4], and occur on banks, ridges and seamounts where nutrient and food supply are comparatively high [5]. Yet, the deep sea is considered to be an energy-limited environment [6], which may explain why CWCs feed opportunistically on a broad range of resources, including dissolved organic matter, phytodetritus and zooplankton [3,7,8].

Despite their occurrence in an energy-limited environment, CWCs are reported to release significant amounts of dissolved and particulate mucus [9,10]. As shown for shallow-water corals [11,12], mucus production by corals may induce a significant energy drain, and so the mucus production by CWCs must have a clear function in terms of fitness and survival. This most likely also holds true for deep-sea corals in warm waters (temperatures exceeding 20°C) but studies on these are so far limited [13,14]. Tropical and sub-tropical shallow-water corals can take advantage of phototrophic carbon fixation through symbiotic algae (zooxanthellae), and hence, mucus production is relatively inexpensive from a metabolic perspective, whilst all aphotic (both warm-water as well as CWCs) live heterotrophically in the dark ocean and depend on organic matter export from the sunlit surface ocean, making mucus production most likely more costly. In this study we focus on CWCs. Both qualitative and quantitative investigations of CWC mucus production have been performed (e.g. [15,16,17]), but the factors driving mucus production have not been entirely assessed. Mucus production is challenging to quantify and often recorded as qualitative observations. As for tropical shallow-water corals [18], mucus production in CWCs appears to occur in response to various stimuli, and studies have reported mucus release by *L*. *pertusa* in response to food stimuli, mechanical and electrical disturbance [16,19], infestation by other organisms [20] and exposure to elevated concentrations of suspended sediment [12,17,21].

A more quantitative and systematic approach to mucus production is needed, but this is hampered by methodological challenges. One important outstanding challenge is the direct observation of mucus production in real-time in ‘undisturbed’ conditions. Until now, the quantity and composition of the mucus produced has been mainly determined from biogeochemical analysis of post-incubation water [9]. However, these biogeochemical data integrate the mucus production over long periods of time, making it difficult to relate production to function. Moreover, the biogeochemical approach cannot be used with certain stimuli (e.g. organic food sources) because these interfere with the biogeochemical analysis. Accordingly, direct microscopic observation of mucus production provides more insight into the functional role of coral mucus. Real-time microscopic imaging of the coral’s polyp surface has recently shown a surface mucus layer several hundreds of μm thick in massive tropical shallow-water corals [22]. Still, such conventional light microscopy is confronted with an important limitation. Because the mucus is transparent and invisible to light microscopy, one can only infer the production and movement of the mucus by tracking opaque particles (such as graphite [19] and activated charcoal particles [22]) that are trapped within the mucus layer.

Here, we employed a novel microscopic technique, digital holographic microscopy (DHM), which for the first time, allowed to make direct microscopic observations of mucus production in the CWC *L*. *pertusa*. The key advantage of DHM is the quantitative nature of the optical phase information that is recorded, which allows the visualization of transparent mucus in aqueous solutions and even enables to distinguish different mucus types. We imaged mucus production in response to various stimuli: (a) exposure to sediment particles and commonly used activated charcoal particles, (b) exposure to the food particle *Artemia salina* as mucus production may be involved in the feeding process [23,24], and (c) exposure to air as this is a common method to ‘harvest’ mucus from shallow-water corals [10,18]. The DHM technique allowed us to observe polyp behaviour, mucus production and identify mucus types at the μm-scale in laboratory incubations. Most importantly, DHM imaging occurred (1) *in vivo*, (2) without using stains and (3) in a non-invasive manner.

## Materials and Methods

### Specimen collection

Colony fragments of *L*. *pertusa* were carefully collected by a ROV (Remotely Operated Vehicle) deployed from the Research Vessel “MS Gunnerus” in the Trondheimsfjord (Norway) on 21 and 24 May 2013. This project was part of a research project from the Research Council of Norway and included the permission to collect and study the protected species *L*. *pertusa* from the Trondheimsfjord. The collection of *L*. *pertusa* fragments was done exclusively for research purposes, involved a minimal number of small colonies and was carried out using a ROV, modified with the front to receive a basket where corals were gently collected one by one to avoid any collateral damage. Coral fragments were first maintained at the nearby biological station of NTNU (Trondheim, Norway), before being transported to the International Research Institute of Stavanger laboratory facilities (IRIS, Mekjarvik, Norway) on 6 June 2013. Here, the corals were kept in containers (60 L) with flow-through seawater conditions (7.5 ± 0.2°C, salinity 33 ± 0.5) and in the dark to reduce fouling. They were fed two to three times a week with a solution of live *Artemia salina* nauplii (a common food source for *L*. *pertusa* kept in laboratory conditions). The experiments in this study were conducted throughout December 2013.

Prior to microscopic imaging, individual *L*. *pertusa* fragments were carefully transferred (while maintained underwater) to smaller DHM-adapted flow-through chambers and left to acclimatize until the polyps extended their tentacles again (at least 1 h). The flow chambers were acrylic cylinders (9.9 cm inner diameter holding approximately 615 mL of water). A continuous flow-through of seawater was maintained with a peristaltic pump set at approximately 10 mL min^-1^, sufficient to ensure circulation of water in the chambers and a renewal of the total water volume within approximately 1 h. The DHM imaging set-up was installed in a temperature-stable working space (17°C), while the running seawater was maintained at a temperature of 7.5 ± 0.2°C. DHM imaging was not carried out in the dark, as *L*. *pertusa* is a deep-sea coral that is known to not be sensitive to light [16,18].

### Imaging technique—digital holographic microscopy

Recent improvements in computing and processing capabilities have enabled the use of digital holography as an imaging tool in a wide range of applications in the life sciences [25,26] but also the marine sciences [27]. One main advantage of DHM over classical bright-field microscopy is its ability to obtain quantitative phase information (see Zetsche et al. [27] for a more detailed review of the DHM technology and the advantages of quantitative phase information for oceanographic applications). In contrast to light microscopy, where only differences in light intensity between the background and object are captured, DHM additionally records the phase information, and this way, enables the visualization of differences in optical path length (OPL) [28]. The OPL is the product of the geometric length of the path a light propagates along, and the refractive index of the medium that it passes through. Light passing through substances, which have the same geometric length but different refractive indices, will correspond to a difference in OPL. The resulting optical path length difference (OPD) is calculated from the phase information recorded by the DHM. In other words, two objects that are both transparent but have a different refractive index (e.g. polymeric substances and mucus extruded by aquatic organisms in seawater) will be distinguished by DHM through the measurement of OPD, but not by traditional light microscopy [29].

**Fig 1:**
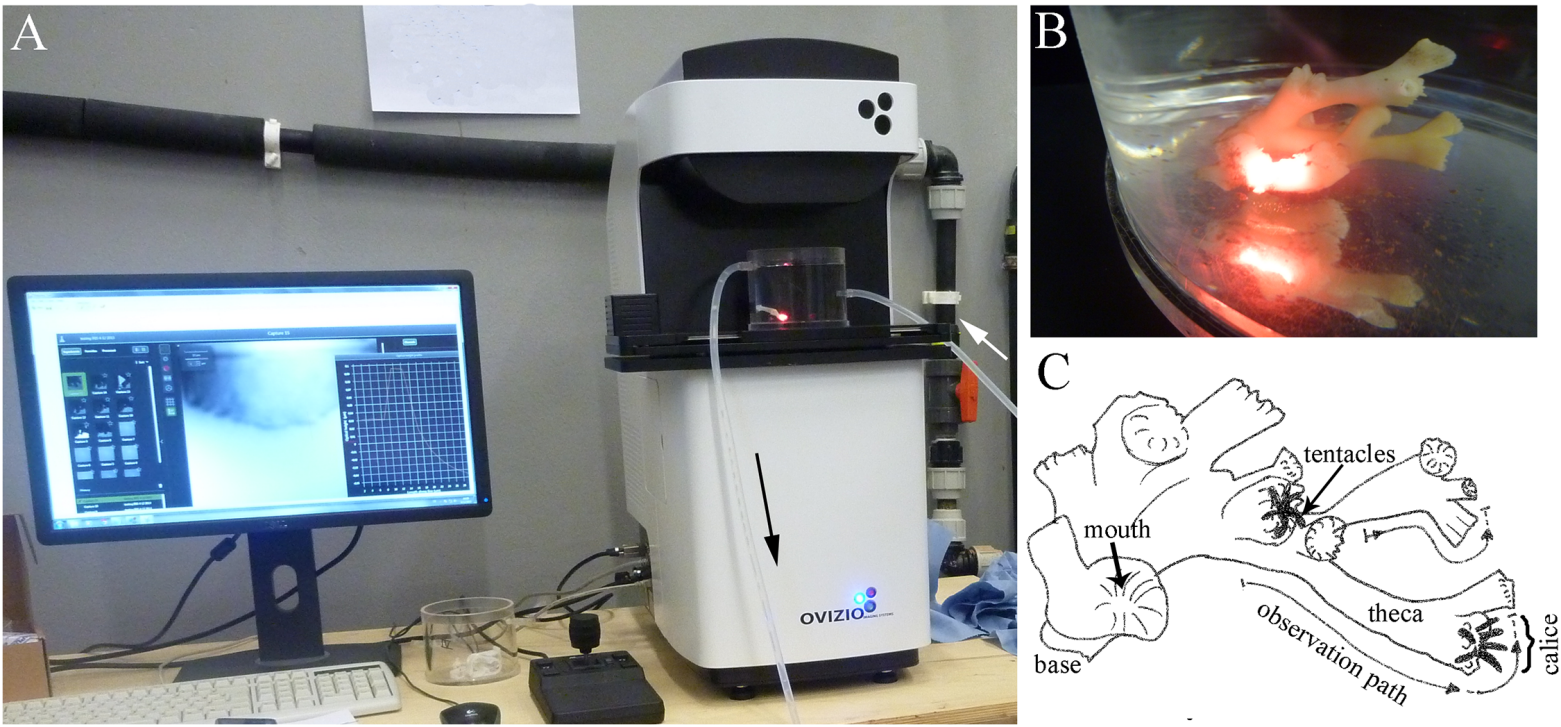
Experimental set-up. A: The imaging set-up includes the digital holographic microscope (D^3^HM) and its computer interface, with a flow chamber placed on the microscope stage. Arrows indicate the direction of the water flow. B: Example of a coral fragment in a flow chamber positioned on the microscope stage ready for imaging. The red light dot is generated by the incident LED beam of the D^3^HM. C: Sketch of one of the corals used in this study, exemplifying the observation paths that were followed before and after exposure to different stimuli.

The digital holographic microscope employed in this study was an oLine D^3^HM (hereafter referred to as D^3^HM) developed by Ovizio Imaging Systems (Belgium), which uses an off-axis self-interference approach based on the principle of differential digital holographic microscopy. Still images and videos were taken and processed with OsOne software (version 3.2), provided with the D^3^HM. In ‘live’ mode the hologram can be directly inspected on a monitor, which was used for the detection of (transparent) substances in real-time. For each captured image, the software provides the source hologram, the light intensity and phase image derived from the hologram, as well as a false-coloured 3D rendition of the quantitative phase information. The human eye has difficulty in seeing minor changes in grey level so that the phase information is more clearly visualized with false colouring. False colours are automatically assigned by the program to the spread of values within one image to enhance the contrast and make differences more visible to the observer. Blues indicate the lowest values of OPD, increasing via greens and yellows to red colours. The D^3^HM was used with a red LED light (630 nm), and a Leica 4x objective. The objective provided a field of view of approximately 1.6 x 1.6 mm and a suitable working distance to the coral polyps to allow imaging of *L*. *pertusa* in the flow chambers and thus in a controlled environment (Fig 1A and 1B).

### Imaging within flow chambers

Individual fragments of *L*. *pertusa* used in the experiment were small, consisting of 9–15 polyps. Polyps were positioned to be within the working distance of the D^3^HM without touching the flow chamber walls or base. Once in position, a sketch was drawn to define appropriate observation paths along the fragment (Fig 1C). Along these paths, the corals were observed in real-time and sequences of still images were captured. Particular areas of interest were video-imaged at frame rates of 1–5 frames s^-1^. In each experiment, at least one full observation path from a polyp’s base to its calice (i.e. the upper oral surface of a polyp) was executed along the theca, and two polyps were imaged per fragment (Fig 1C). Corals were observed in the flow chamber while being exposed to different stimuli as described below. A new sequence of images was taken along the same observation path for each new stressor for comparative purposes. Reactions to the stressors were similar for each of the three fragments used in this study. Photographs of the coral fragments before and after particle exposure were additionally taken using a Panasonic Lumix DMC-FT1.

### Application of stressors

Each experiment was conducted following a similar procedure. After acclimatization in the flow chamber, the initial state of the coral fragment under observation was imaged to define the ‘natural’ background condition before perturbation. Subsequently, on different days, three different coral fragments were exposed to several types of stimuli: (a) suspended particles (activated charcoal, drill cuttings particles), (b) a solution of freshly harvested live *Artemia salina* nauplii, and (c) air exposure.

In the suspension treatment, a solution of either charcoal particles (average grain size ~30 μm) or water-based drill cuttings (fine silt particles <63 μm, predominantly ~20 μm; final solution of 25.38 ± 10.95 mg L^-1^) was added gently with a Pasteur pipette into the flow chamber close to the coral. Imaging commenced immediately after the addition of the stimuli. In the food addition treatment, a solution of *A*. *salina* nauplii was added to the flow chamber in the vicinity of the polyps in a similar manner as the suspended particles. Again, imaging commenced immediately after exposure to the stimulus. To fragment number 1, the suspension of *Artemia* nauplii was applied first, before later applying suspended particles. For fragment numbers 2 and 3, the order of application was reversed. In general, exposure to the different suspensions was separated by at least 2 h. Fragment 3, after the particle additions and a given recovery period, was also exposed for 2 min to the air of the temperature-controlled room (17°C), and subsequently returned to the flow chamber for immediate imaging (i.e. ‘air exposure treatment’).

## Results

**Fig 2:**
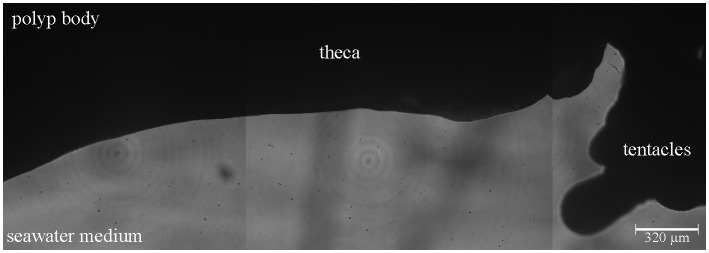
Example of a coral polyp not exposed to stimuli. A merged hologram image is shown for one polyp along the observation path. Dark areas = polyp (opaque to the DHM LED beam), light area = surrounding seawater medium (transparent to the DHM LED beam). No mucus layer or strings were detected along any part of the observation path.

No mucus was observed along the theca or tentacles of coral fragments in control conditions, i.e. maintained in flow-through experimental chambers before exposure to stimuli (Fig 2). However, all applied stimuli induced mucus production.

### Mucus production following particle exposure

**Fig 3:**
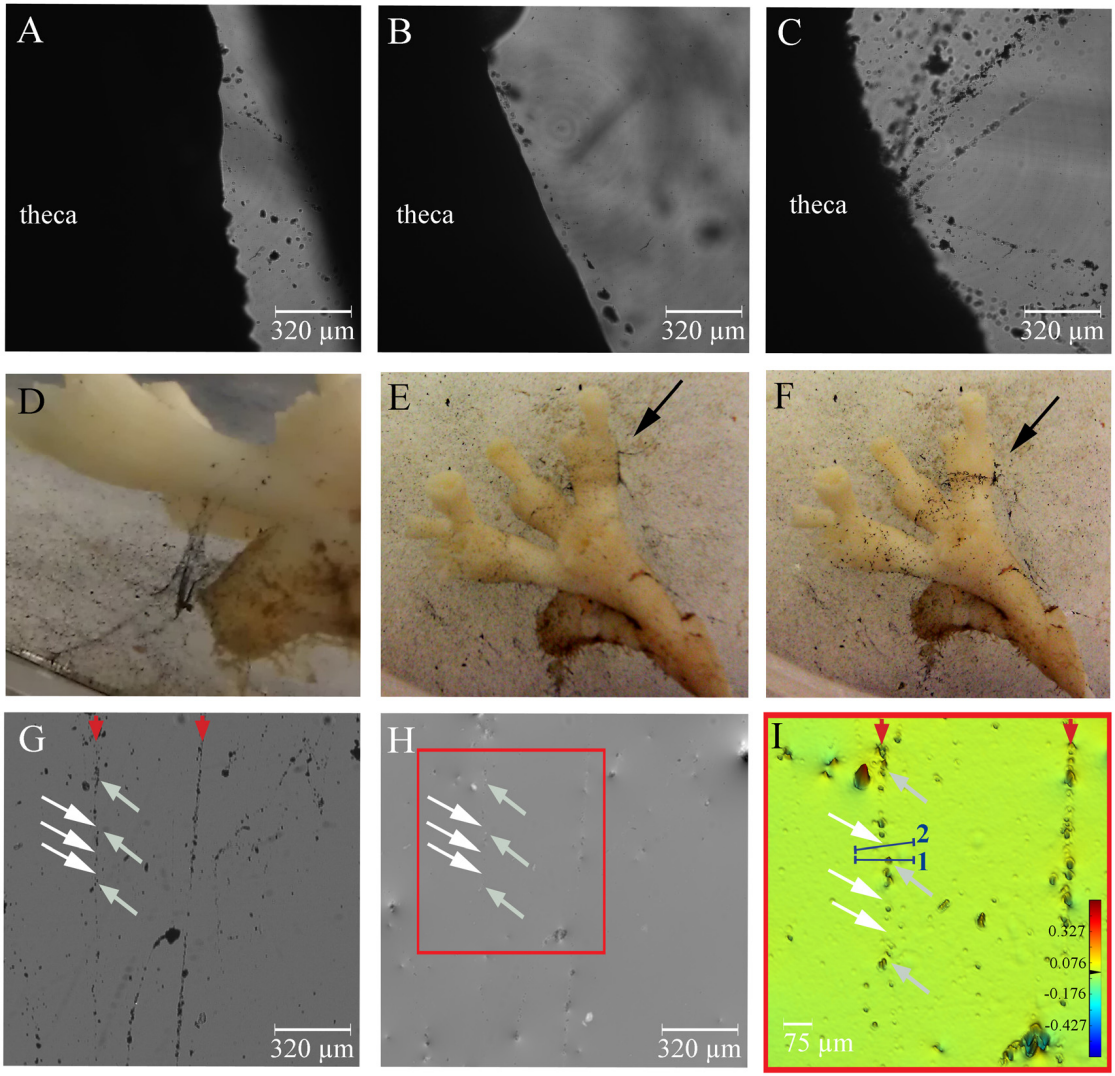
Mucus release after addition of charcoal particles. String-like (A-B) and sheath-like (C) formation of mucus occurs along theca of the polyp. Entrapped activated charcoal particles render the mucus strings visible to the naked eye (D-F). Black arrows in E-F highlight the change in position of particles along the theca. Light intensity image (G), phase image (H) and zoomed in false-colour phase image (I) of the selected area in H of particle-laden strings placed on a glass slide. The position of two particle-laden strings running from the top to the bottom of the image are indicated with red arrows. Grey arrows in G-I point at some of the activated charcoal particles of the left mucus string as examples, whilst the white arrows indicate where mucus is likely to be present connecting particles, although the OPD is not different enough from the surrounding seawater to visualize the mucus with the D^3^HM (I) (i.e. OPD remains at the background level). Blue bars (numbered 1 and 2) depict where profiles of optical path length differences (OPD) were measured. Blue bar ‘1’ crosses a particle of the string, whilst blue bar ‘2’ crosses the mucus string at a position where we would expect the presence of mucus. The scale bar shown in (I) depicts the range in OPD applicable to the image, from its lowest values in the blues to its highest values in the red in μm.

**Fig 4:**
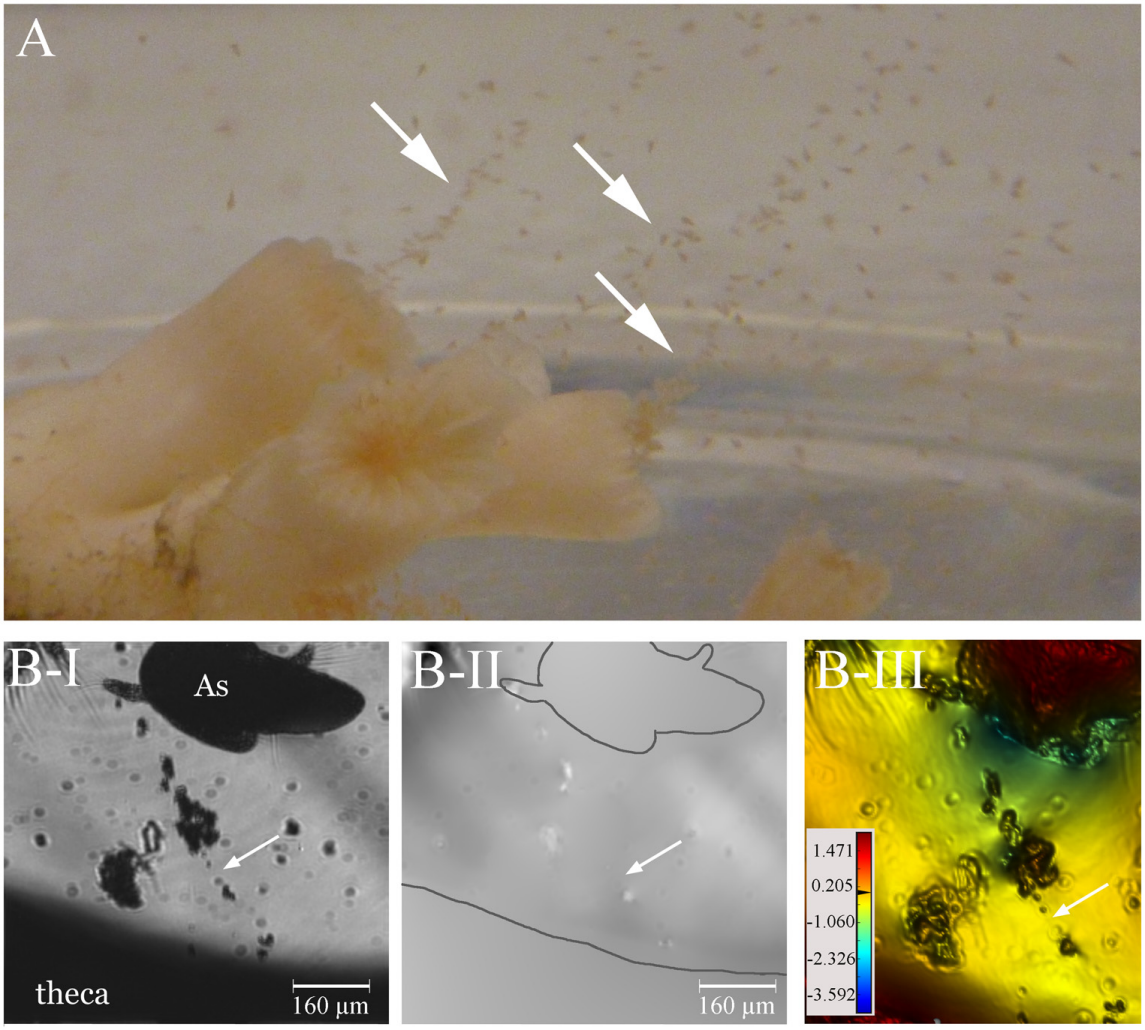
Mucus release after addition of food particles. (A) *Artemia salina* individuals entrapped in mucus strings. The arrows indicate strings extending from the polyp calice towards the surface of the water in the flow chamber. (B) The alignment of trapped *A*. *salina* and small particles clearly hint at the presence of a mucus string (white arrow) in the light intensity image (B-I), yet the optical path length differences (OPD) of these strings were not sufficiently different from the surrounding seawater to visualize them using DHM as shown in the phase image (B-II) and (zoomed in) false-coloured phase (B-III) image, respectively. The polyp and shrimp are outlined in B-II for clarity. The scale bar shown in (B-III) depicts the range in OPD applicable to the image, from its lowest values in the blues to its highest values in the red in μm.

Exposure to suspended particles such as activated charcoal particles or drill cuttings initiated localized mucus production (Fig 3). Both particle types induced the production of string-like and sheath-like structures of various sizes and consistencies that were apparently bound by thin interconnecting secretions (Fig 3A–3C). The presence of mucus strings was often visible with the naked eye due to the entrapment of particles (Fig 3D–3F). Surprisingly however, the mucus that connects the particles could not be visualized with D^3^HM imaging (Fig 3A–3C), not even when strings were placed on a glass slide to remove any potential interferences from the acrylic base of the flow chamber (Fig 3G–3I). In the false-coloured phase image close-up of mucus strings running from the top to the bottom of the image at the positions indicated by the red arrows (Fig 3I), we clearly see the peaks in OPD that are generated by the particles (grey arrows in Fig 3G–3I), yet no connective (mucoid) substance is observed between the particles. This is exemplified in Fig 3G–3I where the white arrows indicate two positions where you would expect a ‘line’ of higher OPD compared to the background running between the particles. Red colours depict the highest OPD values found in Fig 3I, whilst green colours depict OPD of ~0 μm, which dominates this captured image; the OPD of the connections is equal to the background seawater medium. This becomes more clear when OPD profiles are drawn across the particle string (blue bars), first, across a particle of the string (blue bar ‘1’, Fig 3I) as well as an area where you would expect the connective mucoid substance to run along connecting the particles (blue bar ‘2’ in Fig 3I). The profile of the blue bar ‘2’ shows an OPD of 0.008 μm, which is essentially the same as the background noise value. In comparison, a profile drawn across a particle as indicated in Fig 4I with blue bar ‘1’ will measure an OPD of 0.137 μm, an almost 20-fold higher value. These findings indicate that, contrary to the mucus produced following air exposure (OPD of ~0.06 μm, see below), the OPD of the exuded mucoid substance following particle exposure is very low and comparable to that of seawater.

String- and sheath-like mucus was predominantly found in the thecal regions and rarely around the tentacles despite the particle solution being added with a Pasteur pipette along all areas of the polyp. The particle-laden mucus strings and sheaths were generally moving from the polyp’s calice to the base (S1 Video), where they accumulated on the theca (Fig 3E and 3F). Fig 3E shows the position of particles approximately 30 min after they were added close to the polyp (t = 0.5h). Within the next 15 min, particles formed more dense particle- and mucus-laden sheaths, which emerged along the theca of the polyps. Another 15 min later (t = 1h, Fig 3F), clear bands of particle sheaths had accumulated at the base of the polyps and further below at the bases of the various branches of a fragment.

### Mucus production following the addition of *Artemia salina*

**Fig 5:**
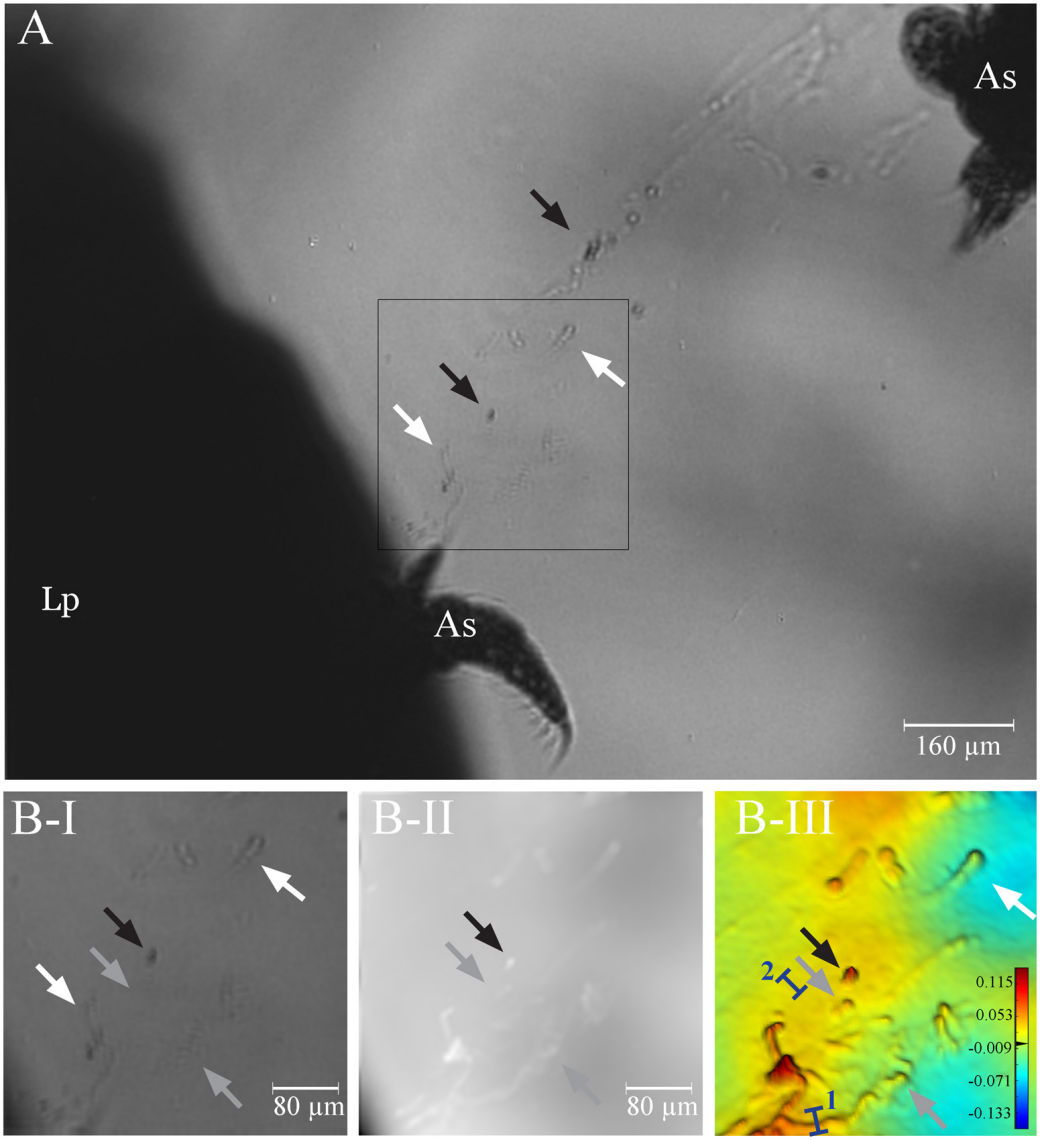
Detailed observation of mucus strings connecting entrapped brine shrimp. (A) Two *Artemia salina* nauplii (As) were connected by mucus strings that contain mucus similar in refractive index to the surrounding seawater, but also particles (black arrows) and mucoid substances having a higher refractive index compared to the surrounding seawater (indicated by white arrows) in the light intensity image. The *A*. *salina* on the left is also attached to a polyp of *Lophelia pertusa* (Lp). (B) A close-up section of the light intensity image (B-I, white and black arrows are in the same position as in A) is already able to discern the presence of some mucoid substance (white arrows), yet it is only in the phase (B-II) and false-coloured phase image (B-III) that we see the full extent of mucus present (see grey arrows). Blue bars (1 and 2) depict where profiles of optical path length differences (OPD) were measured. The scale bar shown in (B-III) depicts the range in OPD applicable to the image, from its lowest values in the blues to its highest values in the red in μm.

The corals displayed various reactions when *A*. *salina* nauplii were added to the flow chambers. No immediate release of mucus (widespread or localized) was observed along the observation paths and tentacles were retracted. After approximately 10 min, however, several *A*. *salina* specimens appeared to be caught and adhered to the coral surface despite efforts to break free, most likely as a result of activated stinging cells (nematocysts), or other cnidae types such as spirocysts that produce sticky webs. *Artemia salina* individuals were also entrapped in mucus strings up to several cm long that were visible by naked eye and predominantly started from the calice (Fig 4A). Like in the ‘particle’ treatment, some of the mucus strings that were formed after the addition of *A*. *salina* nauplii had a refractive index that was not sufficiently different from the surrounding seawater to enable visualization with the D^3^HM (Fig 4BI–4BIII). However, other mucus strings were denser and could be detected (Fig 5), in particular within the false-coloured phase image (Fig 5B-III). The OPD for the profile through the ‘*Artemia’* mucus (blue bar ‘1’, Fig 5B-III) is 0.019 μm and almost 20-fold higher than profile measurements of the surrounding seawater (0.001 μm, blue bar ‘2’), but almost three times lower than for mucus released after air exposure (see next section).

**Fig 6:**
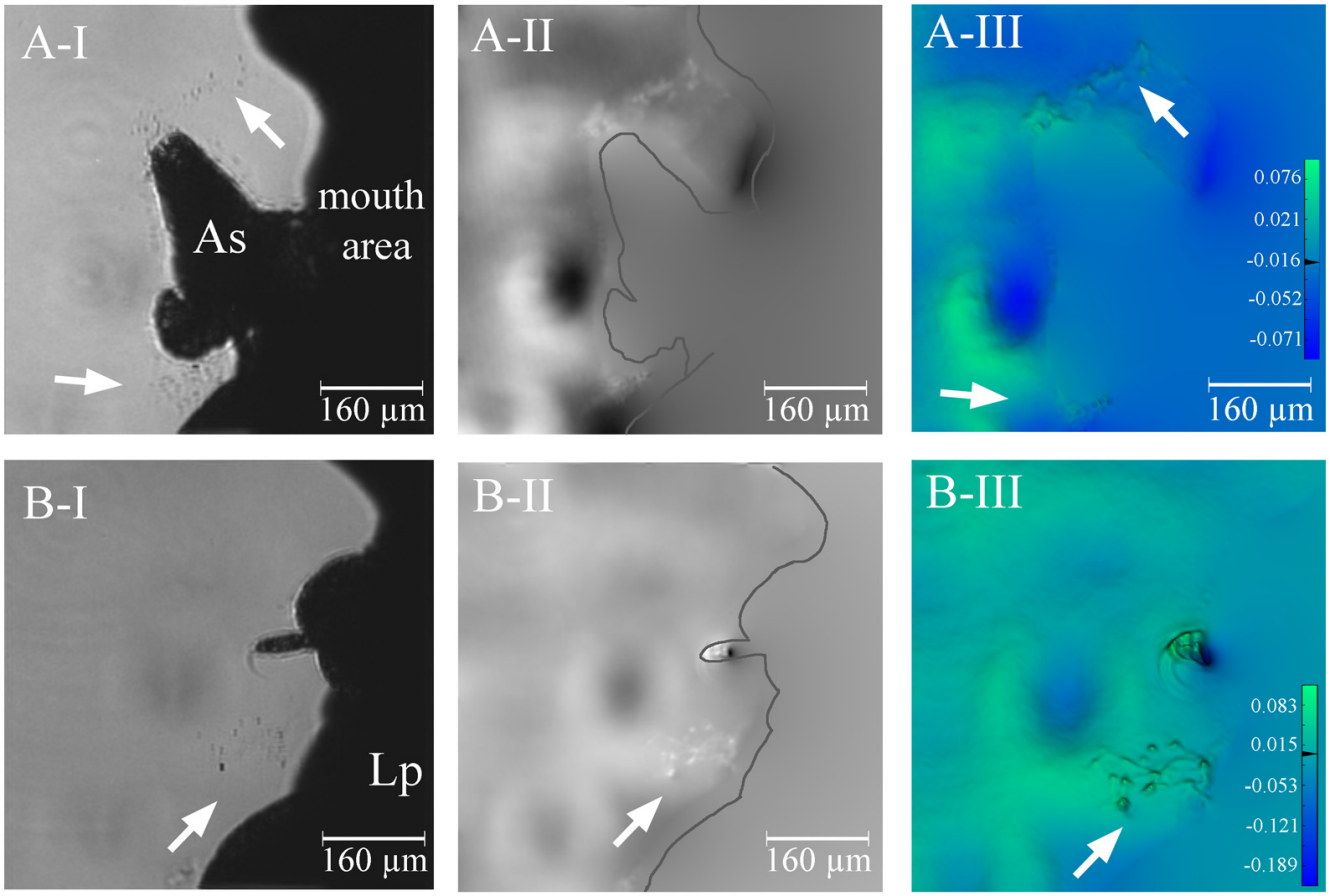
Mucus release associated with digestive processes. (A) An *Artemia salina* nauplius (As) that had been ingested was pushed out of the mouth opening of a *Lophelia* polyp and evidently had mucus attached to it (arrows). (B) Mucus (arrow) associated with the entrapment and potential digestion of the same *A*. *salina* individual now found more directly in the mouth area at a later time point. Subfigures I, II and III are the light intensity, phase and false-coloured phase image, respectively. The polyp and nauplius’ outlines are presented in A-II and B-II for clarity. Note that the phase images here show an uneven background in the seawater medium due to artefacts on the flow chamber base found at a different focus plane. The scale bars shown in (A-III) and (B-III) depict the range in optical path length differences applicable to the images, from its lowest values in the blues to its highest values in the greens in μm.

**Fig 7:**
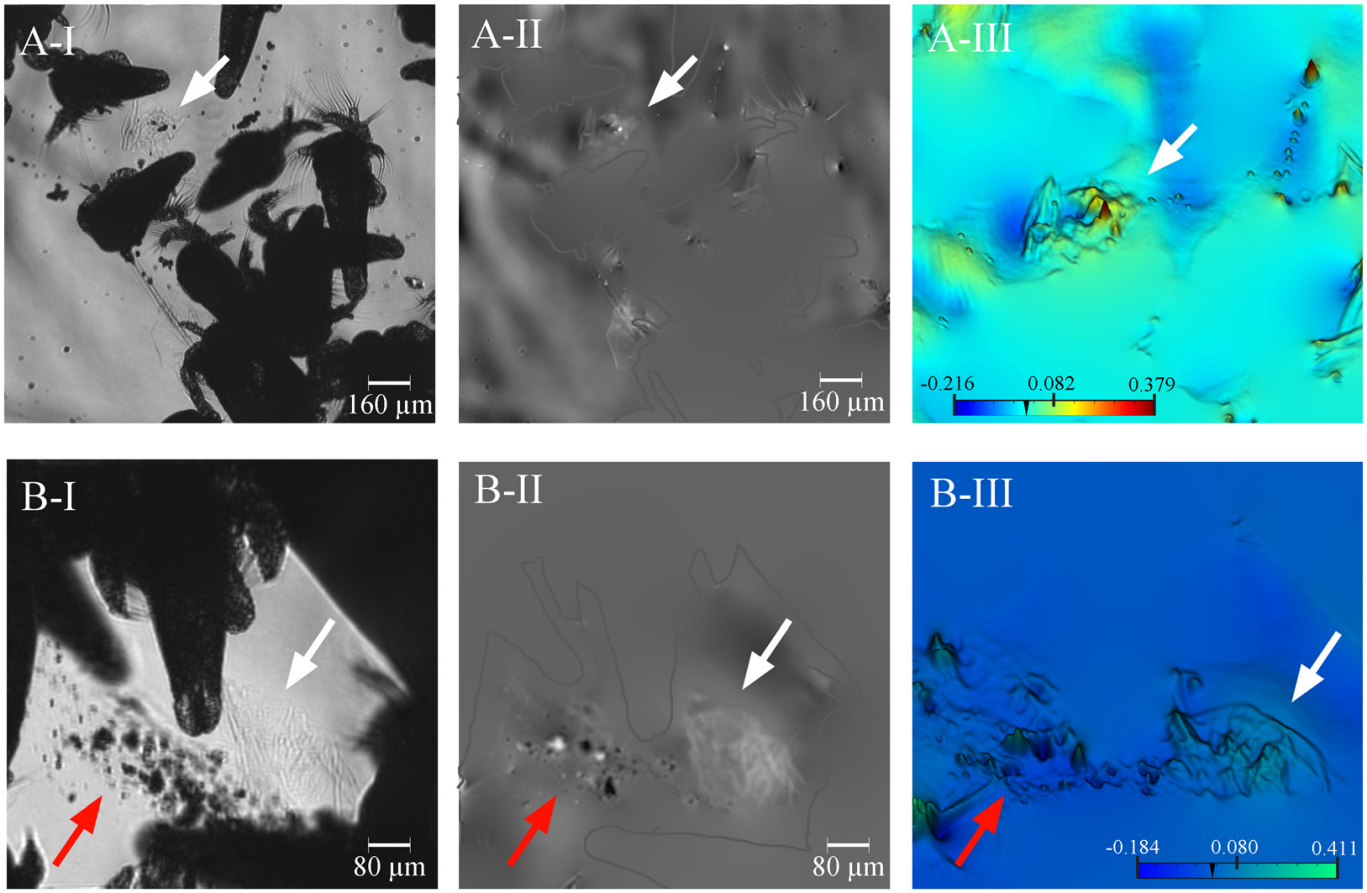
Examples of mucus ‘string balls’. (A) A string ball seen floating among *A*. *salina* individuals. (B) A string ball (white arrow) next to a particle-laden mucus sheath (red arrow). Subfigures I, II and III are the light intensity, phase and zoomed in false-coloured phase image, respectively. The polyp and nauplius outlines are presented in A-II and B-II for clarity. The scale bars shown in (A-III) and (B-III) depict the range in optical path length differences applicable to the images, from its lowest values in the blues to its highest values in the reds (for A-III) and in the greens (for B-III) in μm.

In addition, the D^3^HM images suggest that several other mucus types were formed in the presence of *A*. *salina*. In one example, an individual nauplius was covered with mucus whilst it was being captured and digested (Fig 6A). Mucus of similar optical properties was also observed in accumulations in its direct proximity (Fig 6B, S2 Video) and was presumably exuded in relation to digestive processes. Note how the mucus in Fig 6A-III and 6B-III is clearly visible in the phase images (see arrows) compared to the intensity image (Fig 6A-I and 6B-I) where only individual particles entrapped in the mucus are observed. Finally, more discriminate ‘mucus string balls’, i.e., mucus strings that have been rolled up into ball-like formations, were clearly observed (Fig 7). These mucus string balls were only seen when fragments were exposed to *A*. *salina* and then only in close proximity to *A*. *salina* individuals.

### Mucus production following air exposure

**Fig 8:**
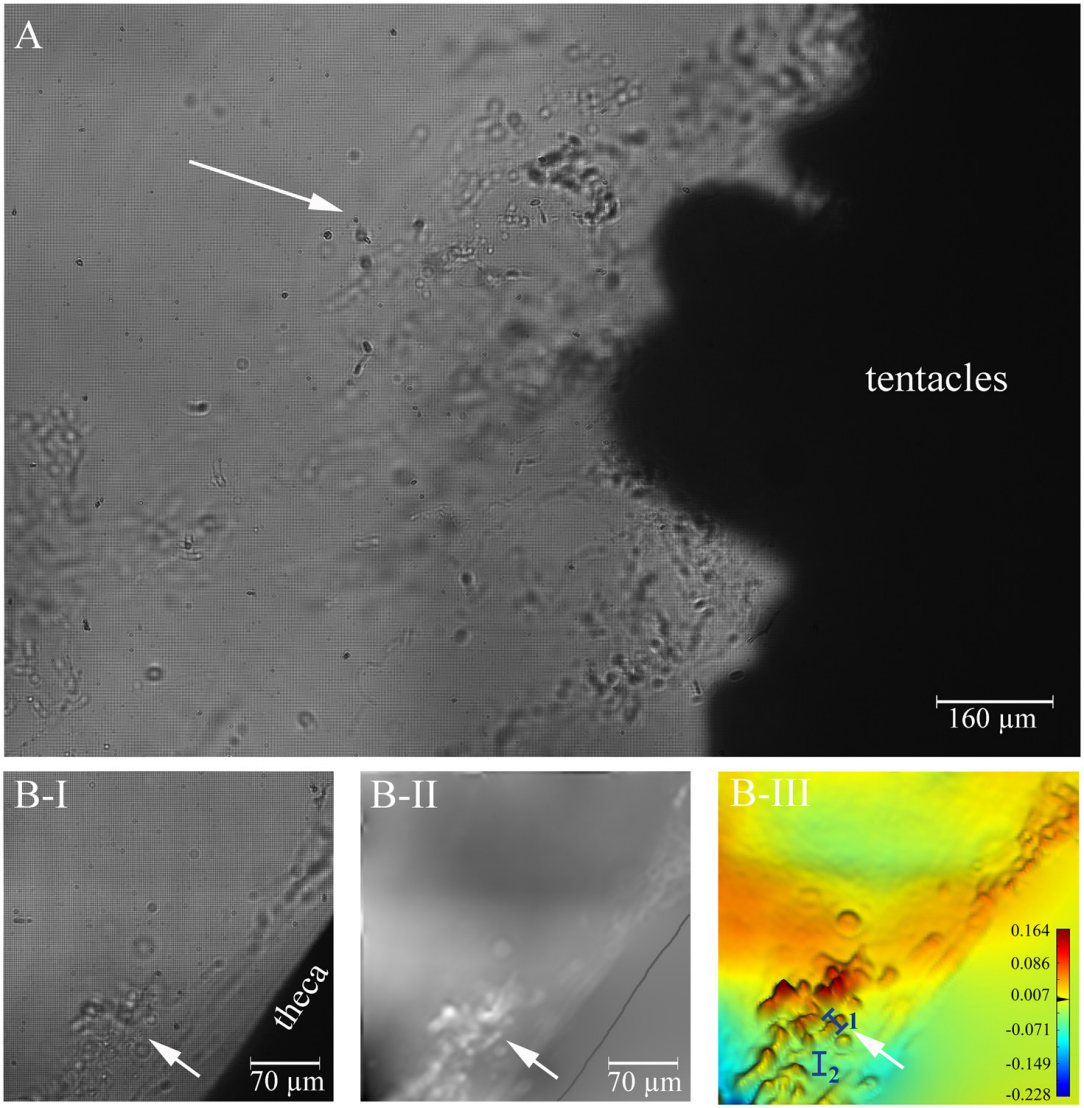
Mucus release after exposure of *Lophelia pertusa* to air and re-introduction into the seawater medium. (A) Hologram image of mucus observed close to the mouth of a polyp. Mucoid material is also shown along a thecal part of a polyp in image series (B) where (B-I) is the initial hologram, (B-II) the phase image, and (B-III) the zoomed-in false-coloured phase image. The white arrow highlights the mucus, which stands out more clearly in the phase information image and false-coloured phase image compared to the hologram. Blue bars (1, 2) depict where profiles of optical path length differences (OPD) were measured. The scale bar shown in (B-III) depicts the range in OPD applicable to the image, from its lowest values in the blues to its highest values in the red in μm.

Mucus was observed by naked eye to be dripping off the coral fragment after it was exposed to air for 2 min. Upon re-introduction into the flow chambers, this released mucus was clearly observed with the D^3^HM. The same response was observed for two other fragments, which were stimulated in parallel, and which had been exposed some weeks before to drill cuttings (data not shown) (Fig 8). Mucus in the chamber water was always seen in close proximity to the coral fragments. In Fig 8A, the hologram image depicts the mucus which was moved by the water flow along the mouth area and tentacles of a *L*. *pertusa* polyp. The advantages of DHM in observing transparent substances become more obvious in the close-up views presented in Fig 8B. The hologram (Fig 8B-I), as observed in live mode on the screen during the experiment, already detects the presence of mucoid substances along the theca of the polyp. However, it is in the subsequent phase image (Fig 8B-II) where these structures are more clearly identified as white areas (see arrow), indicating an increased OPD compared to the surrounding seawater. The profiles drawn across the mucus (blue bar ‘1’ as indicated in Fig 8B-III) gives an OPD of 0.062 μm whilst the profile drawn across the interstitial space (blue bar ‘2”, Fig 8B-III) has an OPD of 0.002 μm.

## Discussion

### Observing mucus release with DHM

Digital holographic microscopy (DHM) offers a novel technique to study the behaviour and physiology of mucus-secreting aquatic organisms in vivo at the micrometre to centimetre scale. Here, the technique was applied for the first time to study the dynamics of mucus release by the CWC *Lophelia pertusa*. In general, no mucus production was observed as long as the corals were not exposed to any form of stimuli. All stimuli examined here (particle suspensions, food addition, air exposure) resulted in mucus release by the coral, but our data show that the type and quantity of the mucus released by *L*. *pertusa* depended on the stimuli. Although all stimuli induced some type of mucus release, some mucus types could be clearly visualized by the D^3^HM technique and other types not. When visualization was not possible, this implies that the mucoid substance had an OPD that is comparable to seawater, i.e. the mucus is delicate and ‘watery’. Other polymeric substances exuded in seawater (e.g. transparent exopolymeric particles and extracellular polymeric substances derived from algae) have been recently observed with the D^3^HM, yet also with a varying degree of success, depending on the refractive index and consistency of the marine gels (E. Zetsche, unpubl. data). The observation that different types of mucus are released by CWCs is important from a metabolic perspective, especially as the production of watery mucus presumably requires less energy expenditure than mucus with a measureable OPD. Larsson et al. [17] already suggested that the energy investment in mucus production must indeed be low for *L*. *pertusa* when exposed to sedimentary particles.

Different mucus types have been noted previously for tropical shallow-water corals [30,31]. Examining the tropical coral *Fungia* sp., Duerden [32] already noted two different types of mucus, a ‘thin watery mucus’ associated with nutritive particles compared to a more ‘shred or membrane-like’ mucus associated with non-nutritive particles. Wild et al. [33] also suggests the presence of a soluble fraction as well as a less-soluble fraction of mucus that are released from *Acropora* sp. that have different effects on the mucus’ role as energy carrier and particle trap. The variability in composition, form and properties of coral mucus has been extensively reviewed for tropical shallow-water corals by Brown and Bythell [18] and more recently, by Bythell and Wild [34], but further studies will need to address this more thoroughly, particularly for CWCs and other deep-sea corals in warm waters such as the Red Sea. In addition, future analyses should consider more thoroughly the role of cnidocysts as a mucus component since these are predominantly composed of mini-collagens [35] and will contribute to the optical as well as physical properties of mucus.

During all our experiments we could not observe the presence of an outer mucus layer along the coral skeleton, as has previously been shown for tropical shallow-water corals [22] and which provide a suitable habitat for a diverse range of symbiotic micro-organisms [36,37]. Molecular studies also describe a specific, presumably symbiotic, bacterial community associated with *L*. *pertusa* [38], and recently, the microbially-mediated processes of nitrogen fixation and de-nitrification were found to be active in *L*. *pertusa* [39]. We do not exclude that a surface mucus layer exists on the epidermis. However, our DHM imaging shows that it must be considerably thinner than the 145 to 700 μm thick layers that have been observed on some massive tropical corals [22]. A strongly reduced outer mucus layer may be one adaptation by which CWCs reduce energy investments in a food-limited environment.

### Particle removal by *Lophelia pertusa*

In their natural environment *L*. *pertusa* can be exposed to higher sediment concentrations due to local high bottom currents, but also higher particle loads where human activities such as offshore drilling operations [40] or bottom fish trawling [41] occur. In accordance with previous studies [16,19,42], the upper parts of the polyps and tentacles of *L*. *pertusa* were freed of particles very rapidly. Our *in vivo* observations indicate that particles do not easily settle onto the tentacles in the first place, and that particles are actively being moved along the tentacles by cilia (S3 Video), as already observed by Gass [42]. We cannot confirm whether mucus was involved in the process of particle rejection mediated by this ciliary movement. When particles settled on the coral’s surface, *L*. *pertusa* tended to trap particles in mucus strings and sheaths as previously documented by Allers et al. [15]. This response was the same for all fragments, irrespective of whether suspended particles were applied first, or with some time delay after the addition of *Artemia*. The same response also occurred in fragments that had been subjected to suspended particle solutions without any *Artemia* addition (data not shown). We observed that mucus release was a rather localized phenomenon, with strings predominantly found along the theca and regions between individual polyps. The strings were, however, moved around by water turbulence and ciliary movement, which led to their compaction and eventual detachment from the corals. Similarly, Lewis and Price [43] mention how converging currents along polyps roll up mucus filaments into bundles trapping particles being driven off the polyp surface. Shapiro et al. [44] recently showed that overall mass transport of nutrients and oxygen in tropical corals is increased through strong vortical flows driven by cilia and we speculate that such vortices may also prevent particle settling on the coral surface. During our experiments we noticed circular motions of particles and mucus balls (S2 and S4 Videos), which suggest that flow patterns with vortices were also driving mucus transport in *L*. *pertusa*. In addition to these small-scale movements, the low settling rate of particles may further be related to the branched morphology of *L*. *pertusa* [15] and ambient currents [45]. *Lophelia pertusa* is preferentially found at locations with elevated bottom currents, which may aid to remove the produced mucus with the entrapped particles.

The tentacles and upper parts of polyps were freed of particles very rapidly, possibly to maintain feeding capacity during high ambient particle concentrations. The particles, often entrained in mucus, that remained attached to the surface eventually accumulated on the tissue-free parts of the skeleton at the base of the polyp (Fig 3E and 3F), which is in accordance with earlier findings [16,17,19]. These particle accumulations may eventually be removed, but complete shedding of accumulated sediment may take up to 9 h [21]. Or, particle accumulations, irrespective of whether originally from natural sediments or human activities (e.g. drill cuttings), may become persistent on the tissue-free (i.e. bare) skeleton [17] and lead to tissue smothering on adjacent living tissue [45].

### Stimulation of mucus release by *Artemia salina*

Cnidocyte firing and tentacle movement in combination with mucociliary feeding is a common feeding mechanism for scleractinian corals [24,46]. Cnidocytes are stinging cells used to capture prey, and *L*. *pertusa* is known to have batteries of these in its tentacle ectoderm [19,47]. Mortensen [16] notes that the capture of food particles occurs via nematocyst adhesion and tentacle movement in *L*. *pertusa*, which may explain the reduced movement of *A*. *salina* as they become intoxicated and get caught along the mucus strings that we observe. No stinging cells were directly observed but most likely this is a result of the low magnification (4x) used in this study, as isolated nematocysts from sea anemones have been successfully visualized with differential digital holographic microscopy technology at higher magnification (40x) (E. Zetsche, unpubl. data).

We observed the entrapment of *A*. *salina* in mucus strands, confirming similar observations by Purser et al. [23]. Furthermore, Fig 5A clearly shows how the mucus strings originate at the mouth of the polyps and are stretched and aligned due to the water flow conditions within the chamber, reinforcing the more general observation for scleractinian corals that mucus is released from the epidermis of the oral disk as polyps initiate their preparatory feeding posture [46]. We also observed distinctive mucus string balls in association with the addition of *A*. *salina*, however, whether these may be related to digestive processes remains unexplained and would require further study. Compared to the mucus strings that formed during sediment exposure, the mucus resulting from exposure to *A*. *salina* seems to show greater compositional variation, with some strings being observable with the D^3^HM and others not. Mucus observed during *L*. *pertusa*’s exposure to *A*. *salina* may have a higher OPD due to a higher content of cnidae in the expelled mucus in response to food exposure and this effect will need to be addressed more clearly in future studies. Nematocysts may have a role in digestion, since some of the venoms are enzymes [48], yet most of the cnidae in the tentacles will be spirocysts, producing sticky webs (S. Strömberg, pers. comm.).

Duerden [30] already noted that differences in the type of mucus produced exist and that the consistency can also change over time in several Hawaiian coral species. The mucus associated with nutritive particles was dissimilar to that associated with sediment removal [32]. For *L*. *pertusa*, observations by Mortensen [16] do not suggest mucus nets to be an important feeding mechanism, however, he does suggest the presence of small amounts of mucus in association with the loss of captured prey items i.e. the presence of mucus associated with digestive processes. This supports our observations of mucus being directly attached to an *A*. *salina* individual that was in the process of being digested (Fig 6). Mucus is therefore involved in the handling and ingestion of food items in *L*. *pertusa*, however, to what extent is a question that will require additional studies.

### Exposure of *L*. *pertusa* to air

Significant mucus production was observed visually and with the D^3^HM when *L*. *pertusa* was exposed to air. Large mucus sheaths were observed, which differed in extent and structure from the mucus that was observed in response to particle exposure and food addition. The air exposure treatment was applied as the last of the various stressors, and we cannot exclude potential cumulative effects. However, the visual appearance of mucus produced was distinctly different compared to the other stress-induced reactions. Despite care to only handle the fragment at its base, a higher OPD may be the result of having a higher presence of the large cnidocysts from the acontia-like free coils in the expelled mucus, given that air exposure is comparable with a predatory attack to the coral, and thus eliciting a defensive reaction. Significant amounts of these cnidae types have been found in the mucus from *L*. *pertusa* held in The Sven Lovén Centre for Marine Sciences (Tjärnö, Sweden) upon air exposure [49], a so far largely neglected aspect, which will also require further investigation in the future. Air exposure is a method that is commonly used to collect coral mucus for microbial and biogeochemical investigation [18, 31]. Consequently, it is questionable whether the mucus collected this way is also representative of mucus associated with ‘routine’ sediment rejection and digestive processes, confirming the suggestion by Brown and Bythell [18] that different types of mucus with different properties may be produced within a coral colony.

## Conclusions

Mucus production and transport by the cold-water coral *L*. *pertusa* was visualized for the first time in real-time at the μm-scale with a state-of-the-art holographic microscope (D^3^HM). Our results demonstrated that *L*. *pertusa* produced different mucus types in response to various stimuli, ranging from mucus strings, sheaths and ‘string balls’. The measurements of OPD profiles with the D^3^HM enabled to quantitatively differentiate between the various mucus types. Mucus release played an important role in particle removal, which occurred predominantly in the form of mucus string formation. Mucus was also involved in prey capture, retention and digestion. Overall, it appears that mucus production of *L*. *pertusa* is adapted to a food-limited environment. Our data show that mucus production by CWCs occurs in response to specific stimuli and that mucus is generally a localized dynamic process. Mucus appears to be treated by CWCs as a precious resource: mucus production is tightly regulated and is induced for specific ‘targeted’ activities (particle removal, feeding). Given the importance of this species for the entire reef ecosystems, further studies using novel observation techniques such as the DHM in combination with other biogeochemical tools, are necessary to confirm our observations and to fully characterize and quantify mucus production to assess the full energy budget of *L*. *pertusa*.

## Supporting Information

S1 Video Movement of particle-laden mucus strings. Activated charcoal particles entrapped in mucus strings are moved along the thecal walls of *Lophelia pertusa* from the mouth area (upper edge) towards the lower parts and eventually base of the polyp (lower edge). (Video was captured at 1 frame s^-1^ and is shown at 2x speed).
(MP4)

S2 Video Mucus release associated with the digestion of the shrimp nauplius *Artemia salina* by *Lophelia pertusa*. First the light intensity information is displayed before the phase information is shown. In the latter, lighter areas clearly depict mucoid substances being released from the mouth area located in the central part of the image. The polyp shown corresponds to the one shown in Fig 7 where an *Artemia* becomes visible after its egestion at a later time point. (Video was captured at 1 frame s^-1^ and is shown in real-time).
(MP4)

S3 Video Cilia along tentacles remove particles. The movement of (activated charcoal) particles along the tentacles of a *Lophelia pertusa* polyp is always towards the tips of the tentacles. (Video was captured at 1 frame s^-1^ and is shown at 2x speed).
(MP4)

S4 Video Vortical movement of particle strings. Along the polyp surfaces particle strings were observed to follow circular motions on several occasions, here exemplified by one such incident. (Video was captured at 1 frame s^-1^ and is shown at 4x speed).
(MP4)

## References

[1] Freiwald A, Fosså JH, Grehan A, Koslow T, Roberts JM. Cold-water coral reefs. UNEP-WCMC, Cambridge, UK; 2004

[2] Roberts J, Davies A, Henry L, Dodds L, Duineveld G, Lavaleye MSS, et al The Mingulay Reef Complex: an interdisciplinary study of cold-water coral habitat, hydrography and biodiversity. Mar. Ecol. Prog. Ser. 2009; 397: 139–151.

[3] van Oevelen D, Duineveld G, Lavaleye M, Mienis F, Soetaert K, Heip CHR. The cold-water coral community as a hot spot for carbon cycling on continental margins: A food-web analysis from Rockall Bank (northeast Atlantic). Limnol. Oceanogr. 2009; 54: 1829.

[4] Cathalot C, van Oevelen D, Cox T, Kutti T, Lavaleye M, Duineveld G, et al Cold-water coral reefs and adjacent sponge grounds: Hotspots of benthic respiration and organic carbon cycling in the deep sea. Front. Mar. Sci. 2015; 2: 37 10.3389/fmars.2015.00037

[5] Mienis F, de Stigter HC, White M, Duineveld G, de Haas H, van Weering TCE. Hydrodynamic controls on cold-water coral growth and carbonate-mound development at the SW and SE Rockall Trough Margin, NE Atlantic Ocean. Deep-Sea Res. Pt. 1 2007; 54: 1655–1674.

[6] McClain CR, Allen AP, Tittensor DP, Rex MA. Energetics of life on the deep seafloor. PNAS 2012; 109: 15366–15371. 22949638 10.1073/pnas.1208976109 PMC3458337

[7] Duineveld GC, Lavaleye MS, Bergman MJ, De Stigter H, Mienis F. Trophic structure of a cold-water coral mound community (Rockall Bank, NE Atlantic) in relation to the near-bottom particle supply and current regime. Bull. Mar. Sci. 2007; 81: 449–467.

[8] Mueller CE, Larsson AI, Veuger B, Middelburg JJ, van Oevelen D. Opportunistic feeding on various organic food sources by the cold-water coral *Lophelia pertusa*. Biogeosciences 2014; 11: 123–133. 10.5194/bg-11-123-2014

[9] Naumann MS, Orejas C, Wild C, Ferrier-Pagès C. First evidence for zooplankton feeding sustaining key physiological processes in a scleractinian cold-water coral. J. Exp. Biol. 2011; 214: 3570–3576. 10.1242/jeb.061390 21993785

[10] Wild C, Mayr C, Wehrmann L, Schöttner S, Naumann M, Hoffmann F, et al Organic matter release by cold water corals and its implication for fauna-microbe interaction. Mar. Ecol. Prog. Ser. 2008; 372: 67–75.

[11] Edmunds P, Davies PS. An energy budget for Porites porites (Scleractinia), growing in a stressed environment. Coral Reefs 1989; 8: 37–43.

[12] Riegl B, Branch GM. Effects of sediment on the energy budgets of four scleractinian (Bourne 1900) and five alcyonacean (Lamouroux 1816) corals. J. Exp. Mar. Biol. Ecol. 1995; 186: 259–275.

[13] Roder C, Berumen ML, Bouwmeester J, Papathanassiou E, Al-Suwailem A, Voolstra CR. First biological measurements of deep-sea corals from the Red Sea. *Sci*. *Rep*. 2013; 3: 2802 10.1038/srep02802 24091830 PMC3789407

[14] Qurban MA, Krishnakumar PK, Joydas TV, Manikandan KP, Ashraf TTM, Quadri SI, et al In-situ observation of deep water corals in the northern Red Sea waters of Saudi Arabia. Deep Sea Res. Part I 2014; 89: 35–43. 10.1016/j.dsr.2014.04.002

[15] Allers E, Abed RM, Wehrmann LM, Wang T, Larsson AI, Purser A, et al Resistance of Lophelia pertusa to coverage by sediment and petroleum drill cuttings. Mar. Poll. Bull. 2013; 74: 132–140. 10.1016/j.marpolbul.2013.07.016 23915980

[16] Mortensen PB. Aquarium observations on the deep-water coral Lophelia pertusa (L., 1758) (Scleractinia) and selected associated invertebrates. Ophelia 2001; 54: 83–104.

[17] Larsson AI, van Oevelen D, Purser A, Thomsen L. Tolerance to long-term exposure of suspended benthic sediments and drill cuttings in the cold-water coral Lophelia pertusa. Mar. Poll. Bull. 2013, 70: 176–188. 10.1016/j.marpolbul.2013.02.033 23510599

[18] Brown BE, Bythell JC. Perspectives on mucus secretion in reef corals. Mar. Ecol. Prog. Ser. 2005; 296: 291–309.

[19] Shelton G. Lophelia pertusa (L.): electrical conduction and behaviour in a deep-water coral. J. Mar. Biol. Assoc. UK 1980; 60: 517–528.

[20] Freiwald A, Wilson JB. Taphonomy of modern deep, cold‐temperate water coral reefs. Historical Biol. 1998; 13: 37–52.

[21] Brooke S, Holmes M, Young C. Sediment tolerance of two different morphotypes of the deep-sea coral *Lophelia pertusa* from the Gulf of Mexico. Mar. Ecol. Prog. Ser. 2009; 390: 137–144.

[22] Jatkar A, Brown B, Bythell J, Guppy R, Morris N, Pearson J. Measuring mucus thickness in reef corals using a technique devised for vertebrate applications. Mar. Biol. 2010; 157: 261–267.

[23] Purser A, Larsson AI, Thomsen L, van Oevelen D. The influence of flow velocity and food concentration on Lophelia pertusa (Scleractinia) zooplankton capture rates. J. Exp. Mar. Biol. Ecol. 2010; 395: 55–62.

[24] Wijgerde T, Diantari R, Lewaru MW, Verreth JA, Osinga R. Extracoelenteric zooplankton feeding is a key mechanism of nutrient acquisition for the scleractinian coral *Galaxea fascicularis*. J. Exp. Biol. 2011; 214: 3351–3357. 10.1242/jeb.058354 21957098

[25] Kemper B, von Bally G. Digital holographic microscopy for live cell applications and technical inspection. Appl. Opt. 2008; 47: A52–A61. 18239699 10.1364/ao.47.000a52

[26] Alm K, El-Schich Z, Miniotis MF, Wingren AG, Janicke B, Oredsson J. Cells and Holograms—Holograms and digital holographic microscopy as a tool to study the morphology of living cells In: Mihaylova E, editor. Holography—Basic Principles and Contemporary Applications. 2013 pp. 335–351. 10.5772/54505

[27] Zetsche E, El Mallahi A, Dubois F, Yourassowsky C, Kromkamp J, Meysman FJR. Imaging-in-Flow: Digital holographic microscopy as a novel tool to detect and classify nanoplanktonic organisms. Limnol. Oceanogr. Methods 2014; 12: 757–775.

[28] Dubois F, Yourassowsky C, Monnom O, Legros J-C, Debeir O, Van Ham P, et al Digital holographic microscopy for the three-dimensional dynamic analysis of in vitro cancer cell migration. J. Biomed. Opt. 2006; 11: 054032-054032-054035. 10.1117/1.2357174 17092181

[29] Minetti C, Callens N, Coupier G, Podgorski T, Dubois F. Fast measurements of concentration profiles inside deformable objects in microflows with reduced spatial coherence digital holography. Appl. Opt. 2008; 47: 5305–5314. 18846168 10.1364/ao.47.005305

[30] Coffroth MA. Mucous sheet formation on poritid corals: An evaluation of coral mucus as a nutrient source on reefs. Mar. Biol. 1990; 105: 39–49.

[31] Wild C, Naumann M, Niggl W, Haas A. Carbohydrate composition of mucus released by scleractinian warm- and cold-water reef corals. Aquat. Biol. 2010; 10: 41–45.

[32] Duerden J. The role of mucus in corals. QJ Microsc. Sci. 1906; 49: 591–614.

[33] Wild C, Huettel M, Klueter A, Kremb SG, Rasheed MYM, Jørgensen BB. Coral mucus functions as an energy carrier and particle trap in the reef ecosystem. *Nature* 2004; 428: 66–70. 14999280 10.1038/nature02344

[34] Bythell JC, Wild C. Biology and ecology of coral mucus release. J. Exp. Mar. Biol. Ecol. 2011; 408: 88–93.

[35] Beckmann A, Özbek S. The Nematocyst: a molecular map of the Cnidarian stinging organelle. Int. J. Dev. Biol. 2012; 56: 577–582. 10.1387/ijdb.113472ab 22689365

[36] Ducklow HW, Mitchell R. Bacterial populations and adaptations in the mucus layers on living corals. Limnol. Oceanogr. 1979; 24: 715–725.

[37] Ritchie K, Smith G. Microbial communities of coral surface mucopolysaccharide layers In: Rosenberg E, Loya Y, editors. Coral health and disease. Springer Berlin Heidelberg; 2004 pp. 259–264.

[38] Neulinger SC, Järnegren J, Ludvigsen M, Lochte K, Dullo W-C. Phenotype-specific bacterial communities in the cold-water coral Lophelia pertusa (Scleractinia) and their implications for the coral's nutrition, health, and distribution. Appl. Environ. Microb. 2008; 74: 7272–7285. 10.1128/AEM.01777-08 PMC2592914 18849454

[39] Mueller CE. The sum is more than its parts: Key species in the functioning of cold-water coral reef communities. PhD Thesis, University of Utrecht. 2014.

[40] Godø OR, Klungsøyr J, Meier S, Tenningen E, Purser A, Thomsen L. Real time observation system for monitoring environmental impact on marine ecosystems from oil drilling operations. Mar. Poll. Bull. 2014; 84: 236–250. 10.1016/j.marpolbul.2014.05.007 24908516

[41] Palanques A, Guillén J, Puig P. Impact of bottom trawling on water turbidity and muddy sediment of an unfished continental shelf. Limnol. Oceanogr. 2001; 46: 1100–1110.

[42] Gass SE. The environmental sensitivity of cold-water corals: Lophelia pertusa. PhD Thesis, Open University. 2006.

[43] Lewis J, Price W. Patterns of ciliary currents in Atlantic reef corals and their functional significance. J. Zool. 1976; 178: 77–89.

[44] Shapiro OH, Fernandez VI, Garren M, Guasto JS, Debaillon-Vesque FP, Kramarsky-Winter E, et al Vortical ciliary flows actively enhance mass transport in reef corals. PNAS 2014; 111: 13391–13396. 10.1073/pnas.1323094111 25192936 PMC4169935

[45] Larsson AI, Purser A. Sedimentation on the cold-water coral Lophelia pertusa: Cleaning efficiency from natural sediments and drill cuttings. Mar. Poll. Bull. 2011; 62: 1159–1168. 10.1016/j.marpolbul.2011.03.041 21529851

[46] Lewis J, Price W. Feeding mechanisms and feeding strategies of Atlantic reef corals. J. Zool. 1975; 176: 527–544.

[47] Neulinger SC, Gärtner A, Järnegren J, Ludvigsen M, Lochte K, Dullo W-C. Tissue-associated “Candidatus Mycoplasma corallicola” and filamentous bacteria on the cold-water coral Lophelia pertusa (Scleractinia). Appl. Environ. Microb. 2009; 75: 1437–1444. 10.1128/AEM.01781-08 PMC2648162 19114511

[48] Nevalainen TJ, Peuravuori HJ, Quinn RJ, Llewellyn LE, Benzie JAH, Fenner PJ, et al Phospholipase A2 in Cnidaria. Comp. Biochem. Physiol. B. Biochem. Mol. Biol. 2004; 139: 731–735. 15581805 10.1016/j.cbpc.2004.09.006

[49] Strömberg S, Östman C. The cnidome and internal morphology of Lophelia pertusa (Linnaeus, 1758) (Cnidaria, Anthozoa). Acta Zoologica (accepted). 10.1111/azo.12164 PMC5363355 28392575

